# Genome sequences of recombinant *African swine fever virus* isolated from domestic pigs in Vietnam

**DOI:** 10.1128/mra.00821-24

**Published:** 2024-11-18

**Authors:** Diep Van Nguyen, Ngoc Thi Nguyen, Duc Van Nguyen, Tham Thi Bui, Tiep Ngoc Tran

**Affiliations:** 1 Avac Viet Nam Joint Stock Company, Hung Yen, Vietnam; Katholieke Universiteit Leuven, Leuven, Belgium

**Keywords:** *African swine fever virus*, ASFV complete genome, ASFV recombination

## Abstract

African swine fever is a highly contagious and devastating disease of pigs. Here, we present the draft genome assemblies of three Vietnamese *African* s*wine fever virus* (ASFV) isolates: rASF1/2-avac02, rASF1/2-avac03, and rASF1/2-avac07. All isolates were closely related to the Chinese recombinant ASFV strains of genotypes I and II.

## ANNOUNCEMENT

*African* s*wine fever virus* (ASFV) is a species belonging to the *Asfivirus* genus of the *Asfarviridae* family. Members of this species have a genome of approximately 170–194 kb, encoding 160–234 open reading frames (ORFs) (1). ASFV strains are classified into 24 genotypes based on the C-terminal sequence of the B646L gene (2). In Vietnam, ASFV belonging to genotype II emerged in 2019 (3). Subsequently, in 2023, recombinant ASFV strains of genotypes I and II were detected based on (partial) Sanger sequencing of the B646L, E183L, and EP402R genes (4). In this study, we analyzed the genomes of three recombinant ASFV isolates.

Three whole blood samples were collected from pigs in Vinh Phuc (rASF1/2-avac02), Bac Giang (rASF1/2-avac03), and An Giang (rASF1/2-avac07) in 2023. The samples were inoculated onto DMAC cell monolayers for 1 hour at 37°C and 5% CO_2_. The cells were then washed twice with 1× PBS and cultured in RPMI-1640 medium supplemented with 10% porcine serum at 37°C in a 5% CO_2_ incubator for 7 days. Then, the infected cells underwent three rounds of freeze-thaw cycles, followed by centrifugation. The viral DNA was extracted from the supernatants using QIAamp DNA Blood Mini Kit (Qiagen, Germany) and used for qPCR (5). The qPCR results revealed that all these samples were positive for both GI and GII genotypes. Paired-end sequencing was performed on an Illumina Novaseq6000 using the TruSeq DNA Nano 350 bp library Kit (Illumina, USA). Raw reads (Table 1) were trimmed using Fastp version 0.23.4 (6). Pig reads were removed by aligning with the pig genome (GenBank accession no. GCF_000003025.5) using Bowtie2 version 2.5.3 and Samtools version 1.15 (7, 8). The Unicycler version 0.5.0 was used for *de novo* assembly, and CheckV version 1.0.3 was utilized for assessing the quality of single-contig viral genomes (9, 10). Genome annotation was conducted with Prokka version 1.14.6 (11). All bioinformatic tools were run with default parameters.

**TABLE 1:** Summary of the genome sequences of three ASFV isolates presented in the study

Isolate name	rASF1/2-avac02	rASF1/2-avac03	rASF1/2-avac07
No. of raw reads	36,512,518	40,701,898	36,789,944
No. of unmapped host reads	6,274,000	7,619,574	3,307,272
Genome size (bp)	185,899	185,326	185,972
GC content (%)	38.58	38.60	38.57
CDS*^a^*	164	163	164
Coverage (×)	2,678	3,437	209
GenBank accession no.	PQ010732	PQ010733	PQ010734
SRA accession no.	SRX25489213	SRX25489214	SRX25489215

^*a*^ CDS, coding sequence.

**Fig 1:**
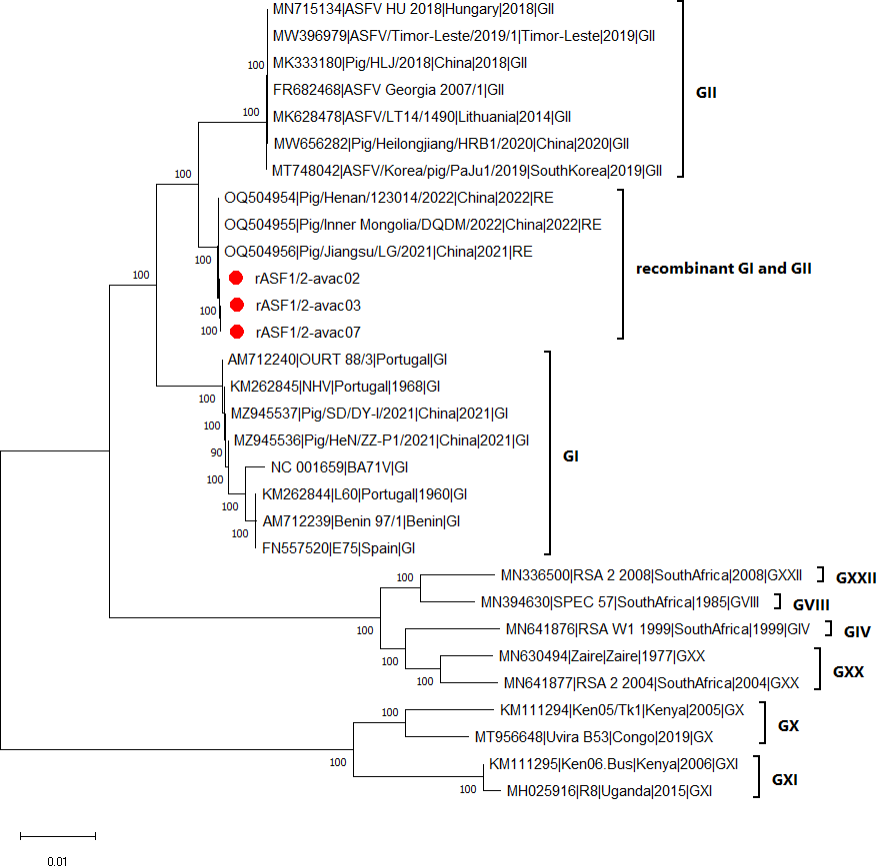
Phylogenetic tree of the *African swine fever virus* was constructed based on the entire genome sequence using the ClustalW alignment algorithm and the neighbor-joining method with 1,000 bootstrap replicates in MEGA11. Bootstrap values above 70 are shown. The scale bar indicates nucleotide substitutions per site. Red dots indicate the sequences analyzed in this study. Other sequences retrieved from GenBank are of genotypes I and II, which are globally distributed, and the recombinant strains between them from China as well as several genotypes restricted to Africa. The accession number and sampling year of each sequence are shown.

The genome sequences of rASF1/2-avac02, rASF1/2-avac03, and rASF1/2-avac07 were 185,899, 185,326, and 185,972 nucleotides long, with average coverages of 2,678×, 3,437×, and 209× and G + C contents of 38.58%, 38.60%, and 38.57%, respectively. Totals of 164, 163, and 164 ORFs were identified for rASF1/2-avac02, rASF1/2-avac03, and rASF1/2-avac07 (Table 1). The genome comparison showed that the genomes of the three isolates share 99.96%–99.99% nucleotide identity with each other and with those of genotype I and II recombinant ASFV strains from China (GenBank accession codes OQ504954, OQ504955, and OQ504956). In the phylogenetic tree, these sequences from Vietnam and China cluster together to form a distinct clade between genotype I and II ASFV clades (Fig. 1), confirming that the three isolates analyzed belong to the recombinant ASFV strain between genotypes I and II.

## Data Availability

The ASFV genome sequences in this report are available in GenBank under accession numbers PQ010732 (rASF1/2-avac02), PQ010733 (rASF1/2-avac03), and PQ010734 (rASF1/2-avac07). The raw reads have been deposited in the NCBI Sequence Read Archive (SRA) database under the accession numbers SRX25489213, SRX25489214, and SRX25489215.

## References

[1] Dixon LK, Chapman D. 2008. *African swine fever virus*, p 43–51. In Mahy BWJ, Regenmortel MHV (ed), Encyclopedia of Virology, 3rd ed. Academic Press, Oxford.

[2] Bastos ADS, Penrith M-L, Crucière C, Edrich JL, Hutchings G, Roger F, Couacy-Hymann E, R Thomson G. 2003. Genotyping field strains of African swine fever virus by partial p72 gene characterisation. Arch Virol 148:693–706. doi:10.1007/s00705-002-0946-8 12664294

[3] Le VP, Jeong DG, Yoon S-W, Kwon H-M, Trinh TBN, Nguyen TL, Bui TTN, Oh J, Kim JB, Cheong KM, Van Tuyen N, Bae E, Vu TTH, Yeom M, Na W, Song D. 2019. Outbreak of African swine fever, Vietnam, 2019. Emerg Infect Dis 25:1433–1435. doi:10.3201/eid2507.190303 31075078 PMC6590755

[4] Le VP, Nguyen VT, Le TB, Mai NTA, Nguyen VD, Than TT, Lai TNH, Cho KH, Hong S-K, Kim YH, Bui TAD, Nguyen TL, Song D, Ambagala A. 2024. Detection of recombinant African swine fever virus strains of p72 genotypes I and II in domestic pigs, Vietnam, 2023. Emerg Infect Dis 30:991–994. doi:10.3201/eid3005.231775 38666642 PMC11060461

[5] Qian X, Hu L, Shi K, Wei H, Shi Y, Hu X, Zhou Q, Feng S, Long F, Mo S, Li Z. 2023. Development of a triplex real-time quantitative PCR for detection and differentiation of genotypes I and II African swine fever virus. Front Vet Sci 10:1278714. doi:10.3389/fvets.2023.1278714 37929278 PMC10620837

[6] Chen S. 2023. Ultrafast one-pass FASTQ data preprocessing, quality control, and deduplication using fastp. Imeta 2:e107. doi:10.1002/imt2.107 38868435 PMC10989850

[7] Langmead B, Salzberg SL. 2012. Fast gapped-read alignment with Bowtie 2. Nat Methods 9:357–359. doi:10.1038/nmeth.1923 22388286 PMC3322381

[8] Danecek P, Bonfield JK, Liddle J, Marshall J, Ohan V, Pollard MO, Whitwham A, Keane T, McCarthy SA, Davies RM, Li H. 2021. Twelve years of SAMtools and BCFtools. Gigascience 10:giab008. doi:10.1093/gigascience/giab008 33590861 PMC7931819

[9] Wick RR, Judd LM, Gorrie CL, Holt KE. 2017. Unicycler: resolving bacterial genome assemblies from short and long sequencing reads. PLoS Comput Biol 13:e1005595. doi:10.1371/journal.pcbi.1005595 28594827 PMC5481147

[10] Nayfach S, Camargo AP, Schulz F, Eloe-Fadrosh E, Roux S, Kyrpides NC. 2021. CheckV assesses the quality and completeness of metagenome-assembled viral genomes. Nat Biotechnol 39:578–585. doi:10.1038/s41587-020-00774-7 33349699 PMC8116208

[11] Seemann T. 2014. Prokka: rapid prokaryotic genome annotation. Bioinformatics 30:2068–2069. doi:10.1093/bioinformatics/btu153 24642063

